# Intrinsic host range of the root-knot nematode *Meloidogyne enterolobii* and virulent *M. incognita* populations

**DOI:** 10.3389/fpls.2025.1668191

**Published:** 2025-10-31

**Authors:** Hemanth Konigopal, Maria R. Finckh, Marc Bailly-Bechet, Etienne G.J. Danchin, Sebastian Kiewnick

**Affiliations:** ^1^ Julius Kühn Institute, Institute for Plant Protection in Field Crops and Grassland, Braunschweig, Germany; ^2^ Faculty of Organic Agricultural Sciences, section Ecological Plant Protection, Universität Kassel, Witzenhausen, Germany; ^3^ INRAE, Université Côte d’Azur, CNRS, Institut Sophia-Agrobiotech, Sophia Antipolis, France

**Keywords:** damage, greenhouse, reproduction factor, quarantine nematode, host plant

## Abstract

The root-knot nematode *Meloidogyne enterolobii* poses a significant challenge in agricultural production systems due to its damage potential and the ability to overcome plant resistance genes, which are effective against other root-knot nematode species. With little plant resistance available, few nematicides still allowed, crop rotation with non- or poor host plants is the only option for managing *M. enterolobii*. As virulence and pathogenicity can vary between *Meloidogyne* populations, determination of the intrinsic host range and pathogenicity of *M. enterolobii* populations is crucial for the implementation of effective management strategies in the future. In greenhouse experiments, the host range and pathogenicity of seven *M. enterolobii* populations were tested on 19 plant species. In addition, two populations of *M. incognita*, virulent against tomato *Mi-1*resistance gene, were included in this study, as they had demonstrated a similar range of reproductive potential and damage compared to *M. enterolobii*. The study revealed that tomato, eggplant, pepper, tobacco, cucumber, potato, bean, melon, sugar beet, yellow mustard, and soybean were good hosts for all tested *Meloidogyne* populations. However, variations in reproduction among populations were observed in carrot, cotton, phacelia, fodder radish, maize, sunflower, and peanut. In rose, none of the *M. enterolobii* populations reproduced (reproduction factor: RF< 0.1). However, virulent *M. incognita* populations allowed some multiplication with RF > 0.1, but below 1.0. Curiously, three *M. enterolobii* populations (M.ent3, 4 and5) showed a lower RF compared to the remaining populations, but were more damaging, resulting in reduced root and shoot fresh weight of the majority of the host plants tested. This is the first study comparing multiple populations of *M. enterolobii*, including the two type populations, from different geographic regions with a large panel of plant species. This study provides crucial information to develop new and sustainable control strategies against the quarantine nematode *M. enterolobii*.

## Introduction

*Meloidogyne enterolobii* was initially described by Yang and Eisenback in 1983 from samples obtained from the roots of *Enterolobium contortisiliquum* (pacara earpod tree) in Hainan province, China. In 1988, a morphologically very similar species, *Meloidogyne mayaguensis*, was described by Rammah and Hirschmann who found this species on roots of *Solanum melongena* (eggplant) in Puerto Rico. Due to the similarities in morphological features, it was long suspected that they were both *M. enterolobii* and based on molecular data Karssen et al. (2012) confirmed that *M. mayaguensis* was as a junior synonym for *M. enterolobii*. Reports in recent years have shown the global distribution and broad host range of *M. enterolobii*, affecting a variety of vegetables, field crops, tree crops, ornamental plants, and weeds (Khanal and Harshman, 2021). Advances in molecular diagnostics have facilitated the accurate detection, identification, and confirmation of *M. enterolobii*, resulting in a significant increase in records of new host plants. In 2022 and 2023 alone, eight new reports were published (e.g. European and Mediterranean Plant Protection Organization, 2022; Anes et al., 2023; Ibrahim et al., 2023; Naveenkumar et al., 2023). *Meloidogyne enterolobii* causes significant yield losses of up to 65% (Philbrick et al., 2020). Its ability to reproduce on many commercial crop cultivars regardless of the source of resistance to other RKN makes crop production difficult (Sikandar et al., 2023). *Meloidogyne enterolobii* is not controlled by resistance in *Capsicum annuum* (*N* gene, Tabasco gene and rootstock ‘Snooker’ carrying the *Mi1* and *Mi3*/*Mi7* genes), *Vigna unguiculata* (Rk gene), *Glycine max* (*Mir1* gene), *Gossypium hirsutum*, *Ipomoea batatas*, *Solanum lycopersicum* (*Mi1* gene) as well as *Solanum tuberosum* (*Mh* gene; European and Mediterranean Plant Protection Organization, 2014; Sikandar et al., 2023).

In the European Union it has been concluded that *M. enterolobii* fulfilled the conditions provided in Article 3 and Section 1 of Annex I to Regulation (EU) 2016/2031 with respect to the Union territory and is listed now in Part A of Annex II to Implementing Regulation (EU) 2019/2072 as Union quarantine pest (Anonymous, 2021). This decision emphasizes that *M. enterolobii* transitioned from an “emerging” species (Elling, 2013) to one of the most detrimental root-knot nematode species worldwide, leaving few options for control (Sikandar et al., 2023). To manage plant parasitic nematode populations, resistant cultivars or crop rotation with non-hosts can be very effective (European and Mediterranean Plant Protection Organization, 2014; Sikandar et al., 2023). Given the declining availability of chemical nematicides and the lack of resistance in major crops with the exception of certain *Psidium guajava* root-stocks (Freitas et al., 2014; Prem et al., 2023), crop rotation with non- or poor host plants will be a primary strategy to manage *M. enterolobii.*

Despite numerous reports of *M. enterolobii* damaging various crops in different geographical regions around the world, the intrinsic host range of *M. enterolobii* populations from different geographical regions and host origins has not been investigated. The intrinsic host range is hereby referred to as the natural or inherent range of plant species that *M. enterolobii* can successfully reproduce on. Therefore, the objective of this greenhouse study was to determine if the extremely wide host range of *M. enterolobii* is intrinsic, therefore determined by its genetic composition or due to other factors such as selection or adaptation. To achieve this, seven *M. enterolobii* populations were used to infect 19 plant species previously reported as major, poor, or non-hosts. In order to maximize the reproduction potential, single egg mass lines of *M. enterolobii* populations, pre-selected for the highest level of virulence were used in these experiments. In this way, declaration of a plant species as non- or poor host due to an intrinsically low reproduction of *M. enterolobii* was avoided. In addition, near isogenic lines from two *M. incognita* populations highly virulent against the tomato *Mi1* gene were included in these experiments as they showed a comparable level of virulence on resistant tomato cultivars when compared to *M. enterolobii*.

## Materials and methods

Greenhouse experiments were conducted under quarantine conditions at the Julius Kühn Institute (JKI) in Braunschweig, Germany. The seven *M*. *enterolobii* populations originated from Africa, Asia and North America, while the two virulent *M*. *incognita* populations originated from Germany (**Table 1**). To determine the host range of these populations, 19 plant species were tested for their potential to serve as a host plant (**Table 2**). All populations were maintained in a greenhouse on tomato cvs. Moneymaker (susceptible) or Phantasia (carrying *Mi1* resistance gene; Volmary GmbH, Münster, DE) throughout this study.

**Table 1:** Populations of *Meloidogyne enterolobii* and virulent *Meloidogyne incognita* from different geographical regions and maintained on tomato under greenhouse conditions, used in this study.

Population	Collection number	Source	Host plant	Species
M.ent1	10.108	Senegal, Africa	Tomato	*M. enterolobii*
M.ent2	C6703	Togo, Africa	unknown	*M. enterolobii*
M.ent3	E1470	China	Pacara earpod tree	*M. enterolobii^a^*
M.ent4	E1834	Puerto Rico	Eggplant	*M. enterolobii^b^*
M.ent5	E8336	Singapore	Cacti	*M. enterolobii*
M.ent8	N01-514-3B	Florida, USA	Guava	*M. enterolobii*
M.ent9	N01-283-14B	Florida, USA	Ornamental plants	*M. enterolobii*
M.inc1		Reichenau, Germany	Tomato root-stock “Beaufort”	*M. incognita^c^*
M.inc2		Reichenau, Germany	Tomato root-stock “Beaufort”	*M. incognita^c^*

^a^ Yang and Eisenback, 1983; ^b^ Rammah and Hirschmann, 1988; ^c^virulent against *Mi1.*

**Table 2:** Plant species used in greenhouse experiments to evaluate the reproductive potential of *Meloidogyne enterolobii* and virulent *Meloidogyne incognita* populations.

Plant species	Common name	Reported host status	Reference	Cultivar used in the study
*Capsicum annum*	Pepper	Major	EPPO Global Database	Yolo wonder
*Cucumis sativus*	Cucumber	Major	EPPO Global Database	Corentine F1
*Glycine max*	Soybean	Major	EPPO Global Database	Primus
*Helianthus annus*	Sunflower	Major	Khanal and Harshman, 2021 Bui and Desaeger, 2021	Peredovik
*Nicotiana tabacum*	Tobacco	Major	EPPO Global Database	White burley
*Solanum melongena*	Egg plant	Major	EPPO Global Database	Clara F1
*Solanum lycopersicum*	Tomato	Major	EPPO Global Database	Moneymaker
*Zea mays*	Maize	Major	Rashidifard et al., 2021	Colisee
*Beta vulgaris*	Sugar beet	Minor	EPPO Global Database	KWS-STD-RES
*Cucumis melo*	Melon	Minor	EPPO Global Database	Cézanne F1
*Daucus carota*	Carrot	Minor	EPPO Global Database	Rote Riesen 2
*Gossypium hirsutum*	Cotton	Minor	EPPO Global Database	n/a
*Phaseolus vulgaris*	Bean	Minor	EPPO Global Database	Scuba
*Rosa* sp.	Rose	Minor	EPPO Global Database	n/a
*Solanum tuberosum*	Potato	Minor	EPPO Global Database	Seresta
*Arachis hypogea*	Peanut	Non-host	EPPO Global Database	n/a
*Phacelia tanacetifolia*	Phacelia	Not tested^1^	Hallmann and Kiewnick, 2015	Balo
*Raphanus sativus* var. *oleiformis*	Fodder radish	Not tested^2^	Hallmann and Kiewnick, 2015	Adagio 56
*Sinapis alba*	Yellow mustard	Not tested^3^	Hallmann and Kiewnick, 2015	Emergo 42

^1^Host status of phacelia not tested for *M. enterolobii*; ^2^Host status of fodder radish not tested for *M. enterolobii*, but resistant cultivars are available against *M. chitwoodi*, *M. hapla*, and *H. schachtii*; ^3^Host status of yellow mustard not tested for *M. enterolobii*, resistant cultivars are available against *H. schachtii*; n/a, not available.

For the experiments, single egg-mass lines were generated from each population. Ten individual females with egg mass were hand-picked and multiplied individually on the tomato cultivar ‘Phantasia’, carrying the *Mi-1* resistance gene. After eight weeks, the reproduction was determined and the line with the highest reproduction factor was selected for further experiments. In addition, species identification for each line was confirmed using Real-time (Kiewnick et al., 2015) and species-specific PCR (Tigano et al., 2010).

To produce inoculum for the experiments, the single egg-mass lines were multiplied twice on tomato cv. Moneymaker. Greenhouse experiments were conducted using plastic pots (11x11x11.5 cm Pöppelmann^®^, Lohne, DE) containing 750ml quartz sand (0.3-1mm) supplemented with slow release fertilizer, Osmocote (1.5g/L). Seeds were germinated in seedling trays and then transplanted into pots. One week after transplanting, plants were inoculated with suspensions containing on average 3800 eggs and second stage juveniles (E+J2) with at least 70% eggs (containing J2s) and 20% J2s. Plants were maintained in a greenhouse during summer at 25 ± 2°C and the winter month at 20 ± 2°C with 16h of light and 8h of darkness. Plants were watered daily and received additional Wuxal^®^ super solution (8:8:6; N: P: K, Hauert MANNA, Nürnberg, DE) once per week. Each host plant species was tested with five replicates and each experiment was conducted twice (summer and winter). Eight weeks after inoculation, the root and shoot fresh weight was recorded and eggs and juveniles (E+J2) extracted from the roots using 0.7% chlorine solution (Stetina et al., 1997) to determine the reproduction factor (RF). Based on the RF values, the host status of tested plant species was defined. Categorization of RF classes was as follows: RF class 0 (non-host) when RF between 0 and ≤0.1; RF class 1 (poor host) when RF >0.1and ≤1; RF class 2 (host) when RF >1 and ≤ 2 and RF class 3 (good host) when RF >2. Although plants with RF >1 are already considered a host (Sasser et al., 1984), the additional class (RF >1 and <2) allows for a more nuanced differentiation of the host plant status.

### Statistical analysis

Statistical analysis was carried out in R (version 4.3.1) software (R Core Team, 2021). Normality of the data was tested using the Shapiro-Wilk test, and homogeneity of variances was checked with the Breusch-Pagan test (P<0.05). Due to the observed heteroscedasticity, data was considered to be non-parametric and analyzed using ANOVA on ranks. Since no interaction between experiments for parameter RF class was observed, data from two experiments were pooled for this variable. The heat map depicting the reproduction factor (RF) of *M. enterolobii* and virulent *M. incognita* populations was created based on four RF class values. Hierarchical clustering of populations was done by ‘hclust’ function and ‘Ward.D2’ method using RF class values. However, as the interactions between experiments were significant for parameters RF, root fresh weight (RFW) and shoot fresh weight (SFW), data from each experiment was analyzed separately (Kruskal-Wallis test and Dunn’s test). The data for RFW and SFW from two experiments were normalized using Z-scores to account for the differences in mean weights between plant species. These normalized scores were then used to illustrate the effects of various nematode populations on different plant species. The phylogenetic data for 19 plant species were obtained from the TimeTree database (Kumar et al., 2022). The classification tree for these plant species, based on their host status with respect to *Meloidogyne* populations, was generated using Ward clustering in R followed by comparison of the topologies for species phylogeny and the classification tree.

## Results

**Figure 1:**
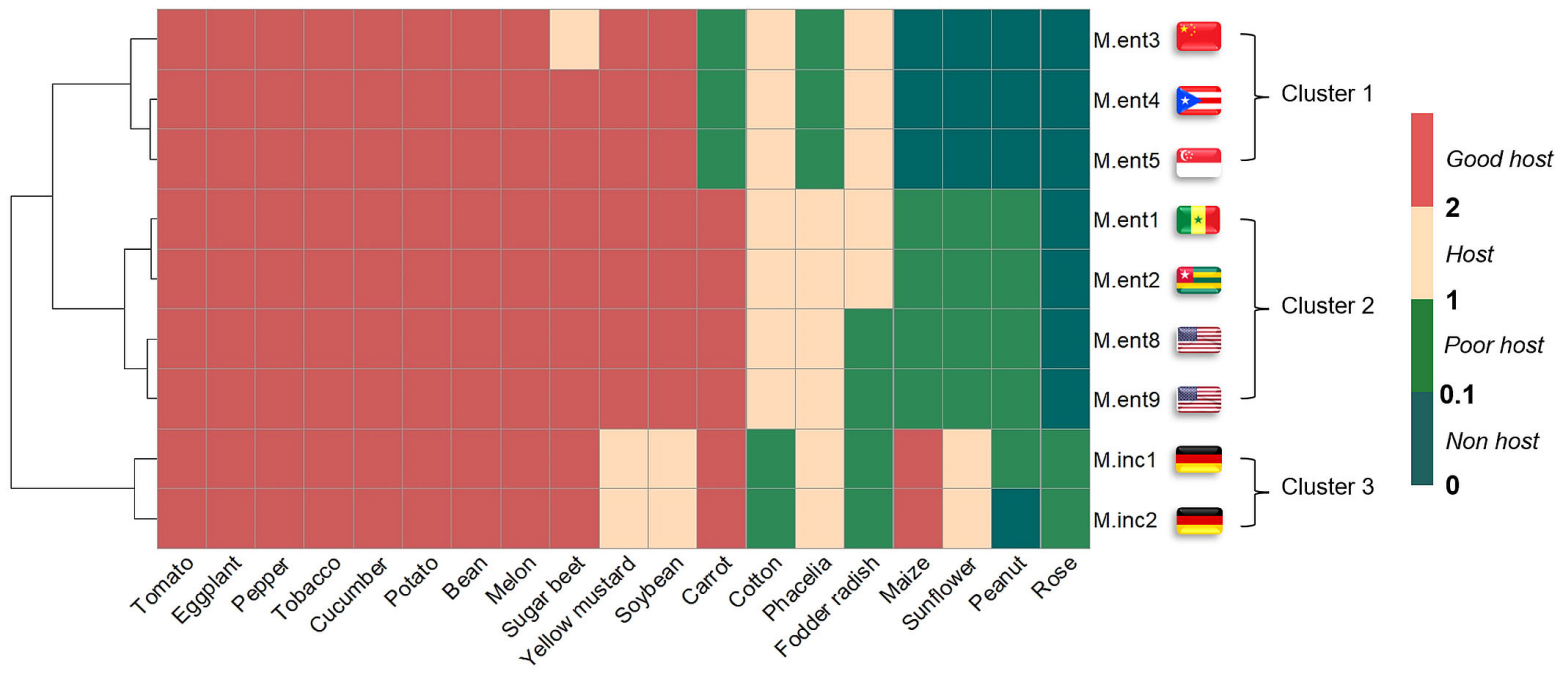
Heat map representing the reproduction factor (RF) of seven *Meloidogyne enterolobii* (M.ent) and two virulent *Meloidogyne incognita* (M.inc) populations across 19 different plant species. The RF class was derived from the RF means and standard deviation of individual treatments, used to depict clustering and the host status for different populations. The RF class was categorised into four groups: RF class 0 (non-host) when RF = 0 and ≤0.1; RF class 1 (poor host) when RF >0.1and ≤1; RF class 2 (host) when RF >1 and ≤ 2 and RF class 3 (good host) when RF >2. Data from two experiments were pooled (n=10) and subjected to non-parametric Kruskal-Wallis test and pairwise Wilcoxon test. The clustering of populations was performed by the ‘hclust’ function and the ‘Ward.D2’ method. Flags represent the geographical origin of populations.

Based on similarities in reproduction factors on 19 host plants, the *M. enterolobii* populations grouped into one cluster containing M.ent3, 4 and 5 and a second cluster with M.ent1, 2, 8 and 9, whereas M.inc1 and 2 formed an outgroup relative to all *M. enterolobii* populations. Across all plant species tested, 13 were hosts for both *Meloidogyne* species, whereas cotton, maize, sunflower and roses significantly differed in their response to the *Meloidogyne* species tested. Phacelia and fodder radish revealed a potential as host or poor host for *M. incognita* and some of the *M. enterolobii* populations (**Figure 1**).

Of the 19 plant species challenged with *M. enterolobii*, 15 were hosts (RF>1) for at least two of the *M. enterolobii* populations, whereas 12 were hosts for all populations (**Figure 1**). The remaining plant species revealed differences in their host status towards the *M. enterolobii* populations. In particular, carrots and phacelia were hosts for populations M.ent1, 2, 8 and 9, but poor hosts for populations M.ent3, 4 and 5. In contrast, fodder radish was a host plant for populations M.ent3, 4, 5, 1 and 2, but a poor host for M.ent8 and 9. Maize, sunflower and peanut were poor hosts for populations M.ent1, 2, 8 and 9 and allowed no reproduction when challenged with populations M.ent3, 4 or 5. None of the *M. enterolobii* populations tested was able to reproduce substantially on roses.

For the two virulent *M. incognita* populations, 15 out of 19 plant species were suitable hosts. Cotton, fodder radish and roses were poor hosts for both populations and peanut proved to be a poor host for M.inc1, but a non-host for M.inc2 (**Figure 1**).

**Figure 2:**
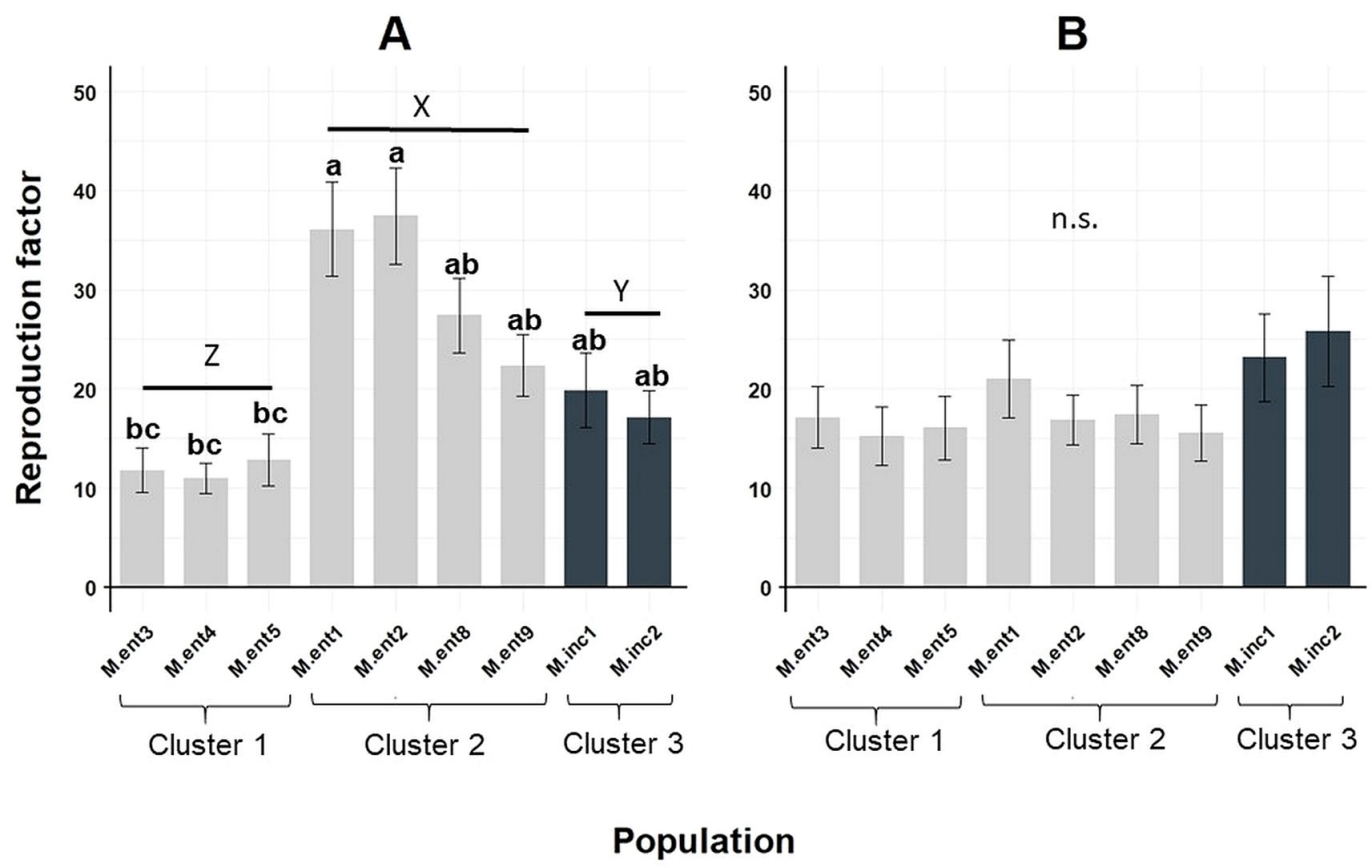
Means of reproduction factor (RF) of seven *Meloidogyne enterolobii* (M.ent) and two virulent *M. incognita* (M.inc) populations averaged across 19 host plant species. Bars indicate the mean RF values ± standard error; **(a)** greenhouse experiment conducted during summer (n=95); **(b)** greenhouse experiment conducted during winter (n=95). Means showing different letters are significantly based on non-parametric Kruskal-Wallis test and Wilcoxon Rank-Sum Test (P < 0.05); Data were LN(X + 1) transformed before analysis, but original non-transformed data are shown. Data pooled per Cluster showing different capital letters are significantly different according to non-parametric Kruskal-Wallis test and Wilcoxon Rank-Sum Test (P < 0.05).

On average, the ensemble of *M. enterolobii* and *M. incognita* populations showed a high reproductive potential across the 19 plant species tested, resulting in RF values ranging from 11 to 37 when greenhouse experiments were performed during summer (**Figure 2a**) and 15 to 25 during winter (**Figure 2b**). RF values for populations M.ent3, 4 and 5 were significantly different to populations M.ent1 and 2 when greenhouse temperatures were higher (**Figure 2a**). Furthermore, data pooled per cluster revealed significant differences between the three clusters (**Figure 2**). Greenhouse experiments conducted during winter resulted in similar reproduction factors across all populations tested (**Figure 2b**).

**Figure 3:**
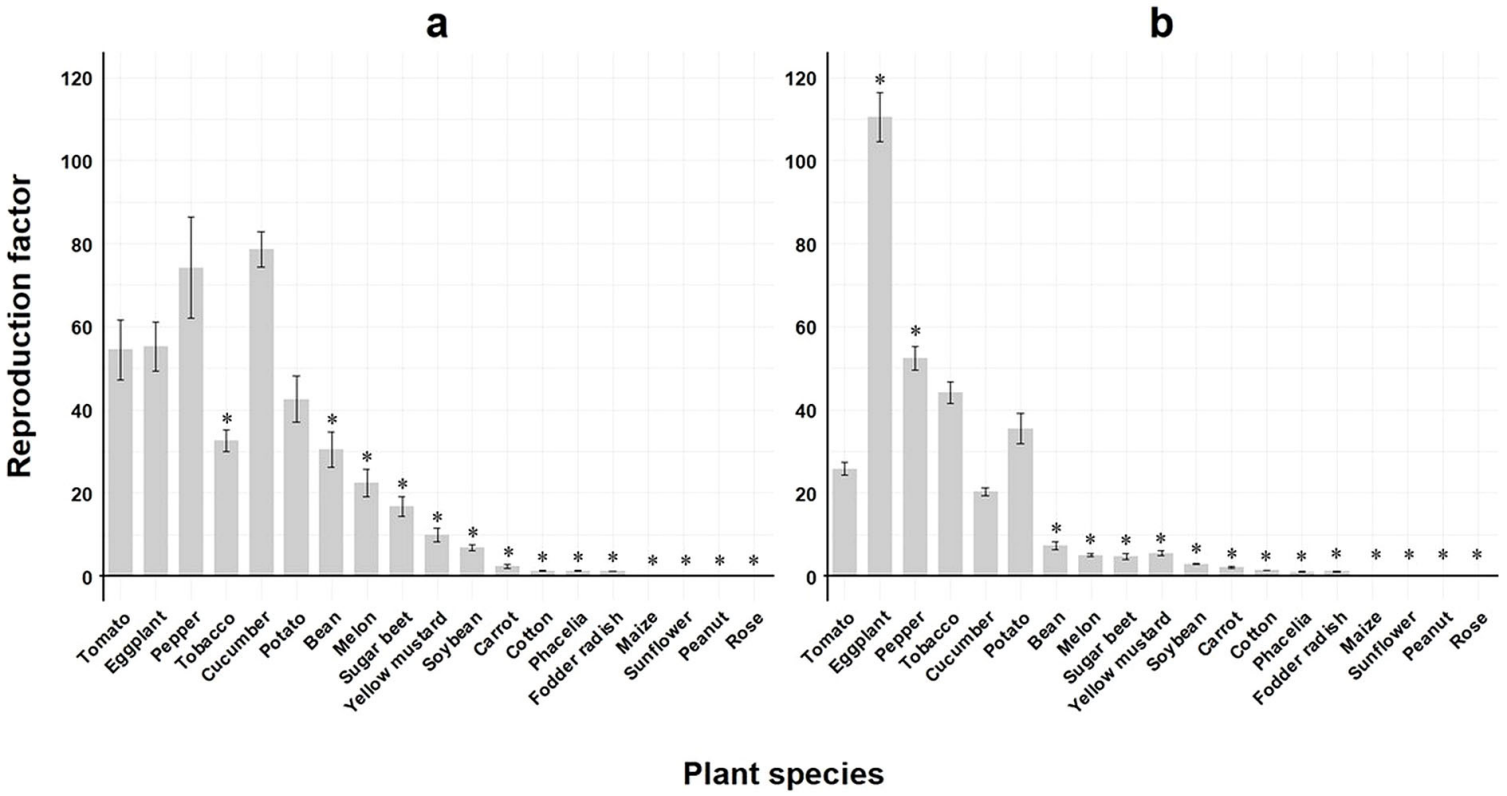
Means of reproduction factor (RF) of seven *Meloidogyne enterolobii* populations across 19 different plant species. Bars indicate the mean RF values ± standard error; **(a)** greenhouse experiment conducted during summer; **(b)** greenhouse experiment conducted during winter (n=35); *significantly different compared to tomato as control according to on non-parametric Kruskal-Wallis test and Dunnet’s t-sided t-test (p< 0.05).

When comparing the reproductive potential across all *M. enterolobii* populations on tomato to the remaining host plant species, all but eggplant, pepper, cucumber and potato showed significantly lower RF values during summer experiments (**Figure 3a**). However, RF values obtained in winter experiments were significantly greater than on tomato for eggplant and pepper, whereas with the exception of tobacco, cucumber and potato, the remaining host plants revealed significantly lower RF values compared to tomato (**Figure 3b**).

**Figure 4:**
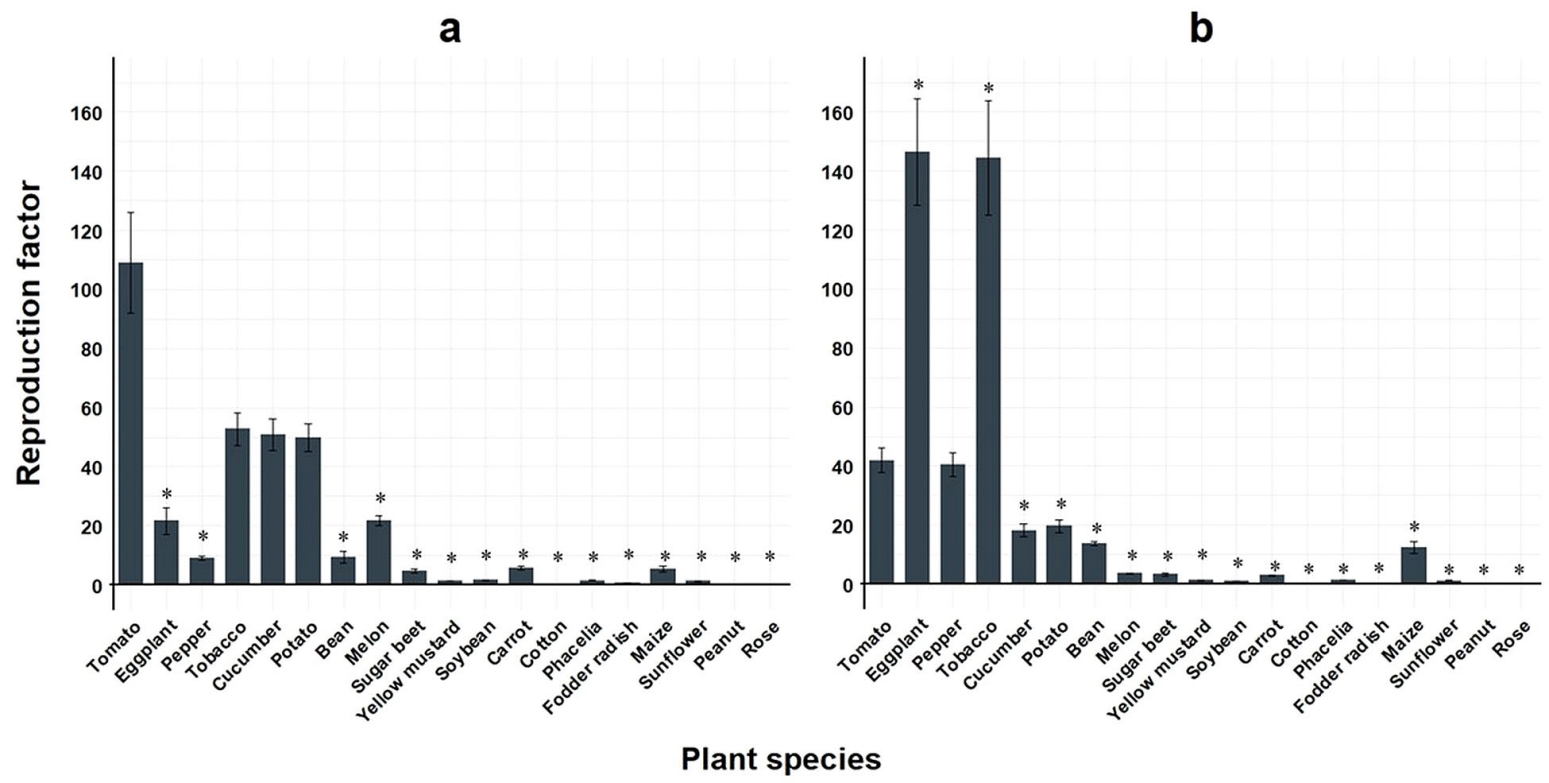
Means of reproduction factor (RF) of two virulent *Meloidogyne incognita* populations across 19 different plant species. Bars indicate the mean RF values ± standard error; **(a)** greenhouse experiment conducted during summer (n=35); **(b)** greenhouse experiment conducted during winter (n=35); *significantly different compared to tomato as control according to non-parametric Kruskal-Wallis test and Dunnet’s t-sided t-test (p< 0.05).

In comparison to *M. enterolobii*, *M. incognita* populations showed significantly lower RF values on 15 out of 18 plant species compared to tomato during summer experiments (**Figure 4a**). During winter experiments, eggplant and tobacco demonstrated a significant, more than 3-fold increase in reproduction compared to tomato, whereas 15 of the plant species showed significantly lower RF values (**Figure 4b**).

**Figure 5:**
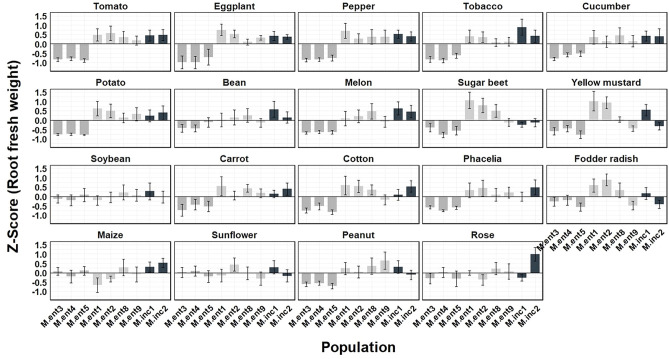
Mean Z score of root fresh weight (g) of 19 plant species challenged with seven *Meloidogyne enterolobii* (M.ent) and two virulent *M. incognita* (M.inc) populations under greenhouse conditions. Data from two experiments were used (n=10). Scoring was achieved using the formula: Z score = (the individual observation - mean of plant species)/standard deviation of plant species. Z = 0: The value is exactly at the mean. Z > 0: The value is above the mean. Z < 0: The value is below the mean. Z > 2 or Z < -2: The value is an outlier. 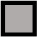 = cluster 1 (population M.ent3, M.ent4 and M.ent5); 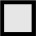 = cluster 2 (population M.ent1, M.ent2, M.ent8 and M.ent9); 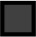 = cluster 3 (population M.inc1 and M.inc2).

**Figure 6:**
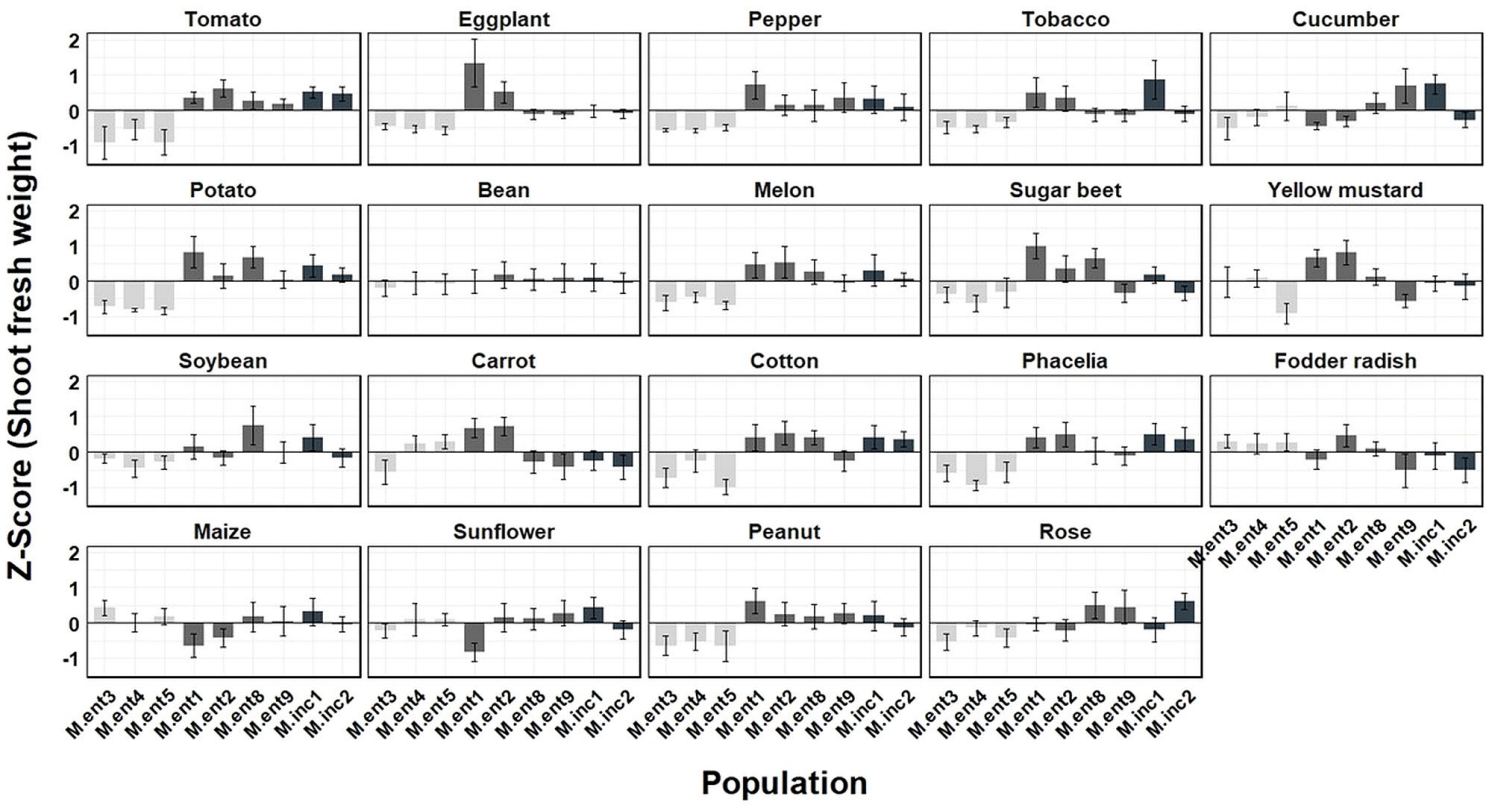
Mean Z score of shoot fresh weight (g) of 19 plant species challenged with seven *Meloidogyne enterolobii* (M.ent) and two virulent *M. incognita* (M.inc) populations under greenhouse conditions. Data from two experiments were used (n=10). Scoring was achieved using the formula Z score = (the individual observation - mean of plant species)/standard deviation of plant species. Z = 0: The value is exactly at the mean. Z > 0: The value is above the mean. Z < 0: The value is below the mean. Z > 2 or Z < -2: The value is an outlier. 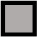 = cluster 1 (population M.ent3, M.ent4 and M.ent5); 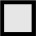 = cluster 2 (population M.ent1, M.ent2, M.ent8 and M.ent9); 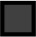 = cluster 3 (population M.inc1 and M.inc2).

To estimate the damage potential caused by different *M. enterolobii* and *M. incognita* populations, Z-Score values were calculated (**Figures 5**, **6**). Based on the average Z-Score for root fresh weight (**Figure 5**), a distinct effect by the *M. enterolobii* populations M.ent3, 4 and 5 was observed across all tested plant species except for soybean, maize, sunflower and rose (**Figure 5**). With respect to shoot fresh weight, 11 out of 19 plant species revealed a negative response due to populations M.ent3, 4 and 5. In contrast, cucumber, bean, yellow mustard, carrot, fodder radish, maize and sunflower revealed no negative effects by populations M.ent 3, 4 and 5. As an exception, rose responded with reduced shoot weight when being challenged by these three *M. enterolobii* populations, although no reproduction was found (**Figures 1**, **6**).

**Figure 7:**
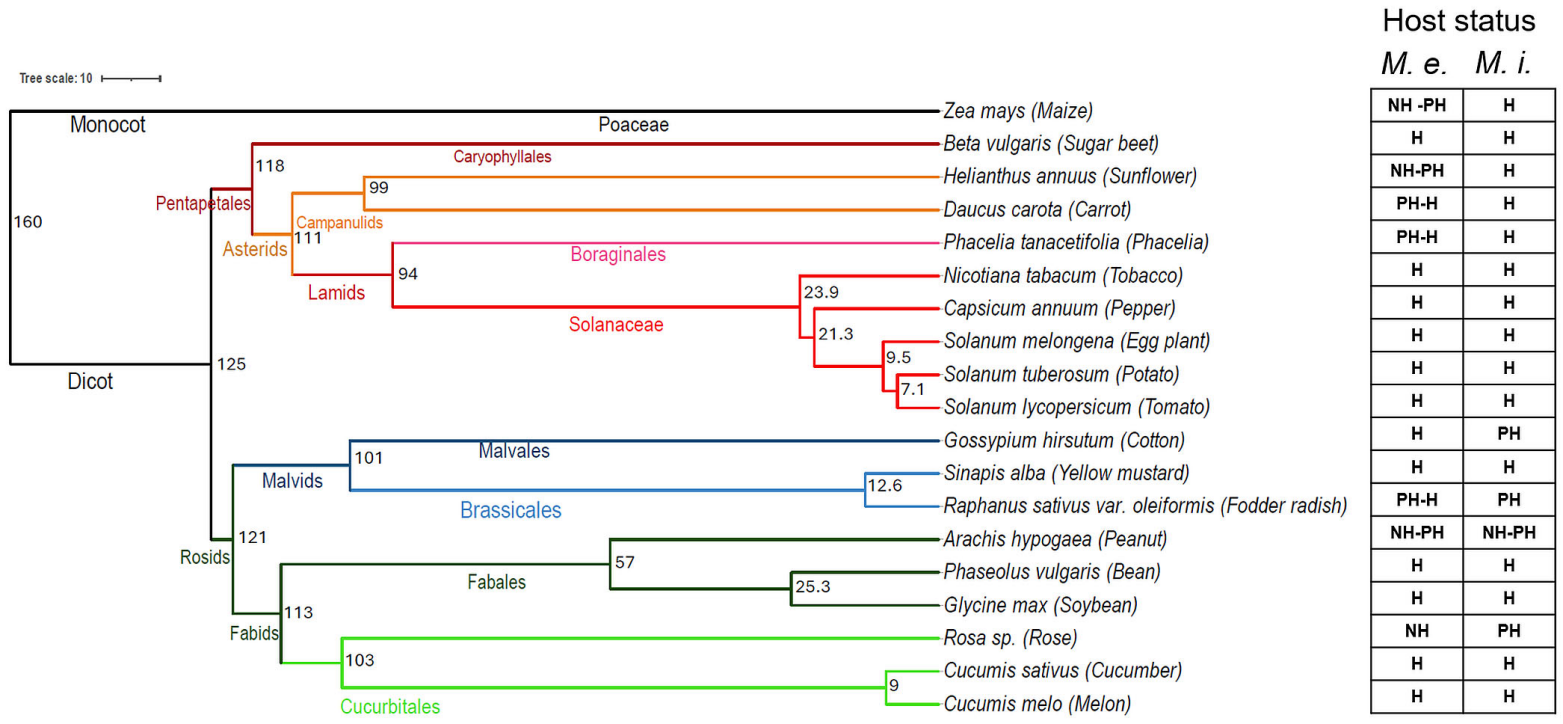
Phylogenetic tree showing the categorization of plants according to the species combined with their range of host status to *Meloidogyne enterolobii* (*M. e.*) and *Meloidogyne incognita* (*M. i.*) The numbers within the classification denote the evolutionary period in million years and the colours indicate the families of the plant species. The dated species phylogeny of 19 crops was obtained from the TimeTree database (Kumar et al., 2022) and the host status classification of plant species was generated using Ward clustering in R; NH, non host; PH, poor host; H, host.

When the phylogenetic distance of tested plant species was compared to their host plant status towards *M. enterolobii* or *M. incognita*, no clear correlation became evident (**Figure 7**). As could be expected, plants of the *Solanaceae* family showed a very similar pattern of host status and grouped together. Cucumber showed a pattern very similar to that of *Solanaceae* although being separated by 125 Million years (Ma) of evolution between the *Solanaceae* and *Cucurbitaceae*. The distantly related monocot maize and the dicot sunflower (160 Ma of divergence) had the same poor and non-host pattern with respect to the *M. enterolobii* populations while they were hosts of *M. incognita*. Similar patterns were observed for peanut and rose (113 Ma), fodder radish and cotton (101 Ma) as well as carrot and phacelia (111 Ma; **Figure 7**).

## Discussion

*Meloidogyne enterolobii*, although regulated as quarantine species in many countries, has become a major challenge for all agricultural production systems worldwide. The increase in the number of reports of new host plants across the world demonstrates the rise of this species from the status “emerging” to a real threat for many main crops on a global scale (Sikandar et al., 2023). Recent reviews documented the increase in reports describing new hosts of *M. enterolobii* where significant damage was observed, in particular crops with resistance to other RKN species (Sikandar et al., 2023).

This study investigated, for the first time, the intrinsic host range of *M. enterolobii* populations from different geographic and host origins. In particular, single egg-mass lines were used to infect a panel of 19 plants species to evaluate the host range of different *M. enterolobii* populations, including the two type populations from China and Puerto Rico (Yang and Eisenback, 1983; Rammah and Hirschmann, 1988). For comparison, we included two highly virulent *M. incognita* populations, with a similar level of damage and reproduction potential (Hallmann and Kiewnick, 2018).

The majority of the plant species tested in this study reacted as expected when challenged with diverse *M. enterolobii* populations. However, a subset of plant species differed in their response and resulted in clustering of the populations M.ent 3, 4 and 5 versus M.ent 1, 2, 8 and 9. The two *M. incognita* populations M.inc1 and 2, obviously belonged to race 2 (Hartman and Sasser, 1985) with tobacco allowing reproduction, but cotton and peanut were poor to non-hosts. Consequently, these two populations formed an additional distinct cluster.

In previous studies, only *M. enterolobii* populations from the same geographical region were compared for virulence and pathogenicity on different host plants or host plant genotypes (Schwarz et al., 2020; Gaudin et al., 2023; Salazar-Mesta et al., 2023), but no or only minor differences were observed.

In our study, we found deviations from published reports for carrot, maize, sunflower and rose. Brito et al. (2007) identified carrot as a poor host when they tested 14 host plants against *M. enterolobii* (syn. *M. mayaguensis*) populations from Florida, USA. Conversely, our study revealed high reproduction rates for populations M.ent1, 2, 8 and 9, which included the two populations originating from Florida (**Table 1**, **Figure 1**). As these populations were most likely obtained from different original host plants, it could explain this different response. The distinct cluster containing both reference populations M.ent3 and 4 as well as population M.ent5 (**Figure 1**) showed only poor reproduction rates on carrot. Consequently, this is the first report of differences in the host range of *M. enterolobii* populations, based on their geographic and host origin. Further discrepancies concerning potential host plants of *M. enterolobii* were found for sunflower and maize. In contrast to Bui and Desaeger (2021); Khanal and Harshman (2021) or Rashidifard et al. (2021), sunflower and maize were non- or poor hosts for all *M. enterolobii* populations tested, despite using similar cultivars. However, in contrast to carrots, these differences were obviously caused by the intrinsic host range of the *M. enterolobii* populations used in this study.

In the year 2008, a consignment of rose rootstocks from China intended for commercial markets was intercepted in the Netherlands due to the presence of *M. enterolobii*. As one consequence rose was listed as a minor host for *M. enterolobii* (European and Mediterranean Plant Protection Organization, 2008). However, in our study, we identified rose as a non-host for all seven *M. enterolobii* populations (**Figures 1**, **3**). This confirms the study by Mendes and Dickson (2016) who demonstrated that *Fortuniana* rose rootstock, which is also used for the European market, was a non-host for all tropical *Meloidogyne* spp., including *M. enterolobii*. No further studies are available confirming the host status of rose toward *M. enterolobii*. Therefore, further research is needed to investigate the host status of roses. However, interceptions of ornamental plants in the Netherlands and Italy confirmed possible routes of introduction for *M. enterolobii* by *Ficus microcarpa* (European and Mediterranean Plant Protection Organization, 2023). These findings suggest that imported horticultural plants, such as rose or *Ficus* spp., are potential sources for introduction into Europe. Often, these imported plants are briefly maintained or multiplied in commercial greenhouses, which are commonly used for growing major crops such as tomato, pepper, or cucumber, before distribution (European and Mediterranean Plant Protection Organization, 2023).

Phacelia, a cover crop commonly grown in temperate regions for weed suppression and green manure as well as yellow mustard and fodder radish were previously identified as maintenance hosts (RF values of 1.0 ± 0.5) for *M. hapla* (Viaene and Abawi, 1998). Resistant cultivars of fodder radish can be used to effectively control *M. chitwoodi*, *M. fallax*, *M. hapla*, and *Heterodera schachtii* (Hallmann and Kiewnick, 2015). Yellow mustard, grown as either an oilseed or green manure crop, is widely cultivated in Europe (Mitrović et al., 2020) with resistant cultivars available to control *H*. *schachtii* (Hallmann and Kiewnick, 2015). Despite their demonstrated neutrality or resistance against other *Meloidogyne* species and *H*. *schachtii*, phacelia, fodder radish, and yellow mustard supported the reproduction of *M. enterolobii* to varying extents.

When reproduction factors were compared across all *Meloidogyne* populations tested, the three *M. enterolobii* populations M.ent3, 4 and 5 stood out as they showed the lowest reproduction during the summer experiments when temperatures averaged 25 ± 2 °C. Greenhouse experiments conducted during winter showed no differences in reproduction. However, *M. incognita* reproduction was not different from *M. enterolobii* and even greater compared to the above mentioned three populations in the summer experiments. This contradicts previous findings where *M. enterolobii* always showed higher reproduction rates compared to other tropical *Meloidogyne* species (Kiewnick et al., 2009). The effect of temperature on the reproduction of *Meloidogyne* spp. is well documented and therefore expected (Wong and Mai, 1973; Greco and Vito, 2009; Dinardo-Miranda and Fracasso, 2010; Fernandez et al., 2014; Daramola et al., 2021). In contrast, the observed host plant status did not differ between experiments, but in some cases reduced root damage resulted in increased RF values. Overall, the results confirm the findings by Velloso et al. (2022) who showed high reproduction rates for both *M. enterolobii* and *M. incognita* under varying temperature regimes. For few hosts, such as eggplant, the lower greenhouse temperatures (20 ± 2°C) allowed for high reproduction rates of *M. enterolobii* and *M. incognita*, respectively (**Figures 3b**, **4b**).

For some host plant/*Meloidogyne* population combinations, positive Z-scores for root fresh weight were observed (**Figure 5**) as nematode infection stimulates plant growth and the development of secondary roots and galls despite supporting large nematode populations (Tandingan et al., 1996; Parveen, 2006; Guarneri et al., 2022). However, the populations M.ent3, 4 and 5 stood out as they clearly affected the root and shoot fresh weight of several of the tested plant species negatively (**Figures 5**, **6**). As these three populations formed a cluster based on their host range (**Figure 1**), it might indicate a correlation between the intrinsic host range of a given population and its damage potential.

The phylogenetic analysis of the host plant species tested in this study demonstrated the wide reproductive potential of *M. enterolobii* populations on crops with evolutionary distances of up to 160 million years. No correlation was found between the evolutionary distance of plant species and their status as host plant to *M. enterolobii* (**Figure 7**). These findings support the wide host range, varying levels of aggressiveness, and diverse host compatibility of *M. enterolobii*. Few plant species covering the full range of phylogenetic distances such as maize, sunflower, and cotton were able to separate *M. enterolobii* from *M. incognita*. Overall, these findings confirm the wide range of host plant species that *M. enterolobii* is able to reproduce on and cause significant damage. As its intrinsic host range is obviously not restricted to a certain group of closely related plant species it explains the constantly increasing number of reports of new host plant species around the globe (Sikandar et al., 2023).

In conclusion, this is the first study investigating a collection of *M. enterolobii* populations from different geographical and host origins for their intrinsic host range with 19 different host plant species. We confirmed the wide host range of *M. enterolobii* which is comparable to *M. incognita*, currently described as the root-knot nematode species causing the highest economic damage worldwide (Jones et al., 2013). Based on the presented results, current prediction models (e.g. Pan et al., 2023) considering only the impact caused by abiotic, but not biological factors, should be adjusted accordingly. This will lead to predicting wider suitable areas for the spread and establishment of *M. enterolobii* in context of global warming, which has resulted in a northward migration of previously undetected ‘tropical’ RKN species (Gerič Stare et al., 2018). Based on the obtained results, farmers will have few options to use poor- or non-host plants to manage nematode populations once *M. enterolobii* is introduced into Europe. Therefore, further studies on suitable intercrops are needed in support of farmers, as plant resistance is not yet available to control *M. enterolobii*. Discrepancies between published reports on host plants and this study indicate the intrinsic potential of *M. enterolobii* to multiply on all mayor crops worldwide although the use of single eggmass lines potentially have restricted the range. Recently, a high-quality genome assembly of *M. enterolobii* for the type population from Puerto Rico has been published (Poullet et al., 2025). This constitutes a reliable resource for within- and between-species comparative genomics and for the identification of genomic variations in relation with the host range of this quarantine nematode species in support of plant health and to develop new measures for control.

## Data Availability

The raw data supporting the conclusions of this article will be made available by the authors, without undue reservation.
